# Management and Treatment of Human Lice

**DOI:** 10.1155/2016/8962685

**Published:** 2016-07-27

**Authors:** Abdoul Karim Sangaré, Ogobara K. Doumbo, Didier Raoult

**Affiliations:** ^1^Research Unit on Emerging Infectious and Tropical Diseases (URMITE), UMR CNRS 7278, IRD 198, INSERM 1095, Faculty of Medicine, Aix-Marseille University, 27 boulevard Jean Moulin, 13005 Marseille, France; ^2^Epidemiology Department of Parasitic Diseases, Faculty of Medicine and Odonto-Stomatology, Faculty of Pharmacy (MRTC/DEAP/FMOS-FAPH) UMI3189, University of Sciences, Techniques and Technologies of Bamako (USTTB), Bamako, Mali

## Abstract

Of the three lice (head, body, and pubic louse) that infest humans, the body louse is the species involved in epidemics of louse-borne typhus, trench fever, and relapsing fever, but all the three cause pediculosis. Their infestations occur today in many countries despite great efforts to maintain high standards of public health. In this review, literature searches were performed through PubMed, Medline, Google Scholar, and EBSCOhost, with key search words of “*Pediculus humanus*”, “*lice infestation*”, “*pediculosis*”, and “*treatment*”; and controlled clinical trials were viewed with great interest. Removing lice by hand or with a lice comb, heating infested clothing, and shaving the scalp were some of the oldest methods of controlling human lice. Despite the introduction of other resources including cresol, naphthalene, sulfur, mercury, vinegar, petroleum, and insecticides, the numbers of lice infestation cases and resistance have increased. To date, viable alternative treatments to replace insecticides have been developed experimentally* in vitro*. Today, the development of new treatment strategies such as symbiotic treatment and synergistic treatment (antibiotics + ivermectin)* in vitro* has proved effective and is promising. Here, we present an overview on managing and treating human lice and highlight new strategies to more effectively fight pediculosis and prevent resistance.

## 1. Lice and Their Public Health Impact

### 1.1. Overview

Lice have been parasites of humans for thousands of years and differ according to their habitat on the host [1]. The three sucking lice that infest humans are the body louse (*Pediculus humanus humanus*), the head louse (*Pediculus humanus capitis*), and the pubic or “crab” louse (*Pthirus pubis*) [2]. Body lice and head lice harbor the same endosymbiotic microorganism that seems to be essential for the production of nutritional components such as the B vitamins lacking in host feedings [3, 4]. In 2006, Sasaki-Fukatsu et al. were the first to describe the phylogenetic placement of the primary endosymbiont of human body lice; they identified this endosymbiont as a *γ*-proteobacterium and named it* Candidatus* Riesia pediculicola [5].

Each year, louse infestations still affect hundreds of millions of people worldwide [6], 6 to 12 million children in the United States annually [7]. Analysis of data on the global incidence of pediculosis has shown that this remains a major health problem in many countries [8]. Thus, the head louse is prevalent in all countries, and outbreaks have been described at all levels in society [9]. However, children of primary school age constitute the largest group of people affected [10]. Approximately 6–12 million cases of infestation occur each year in the United States among children 3–12 years old [11]. Infestation may be increased in school children with more siblings and of lower socioeconomic group [12]. The pubic louse is usually a sexually transmitted organism, although atypical locations such as eyebrows and eyelashes have been reported [9, 13]. The body louse lives in clothes and multiplies when cold, promiscuity, lack of hygiene, and war are present. Its prevalence also reflects the socioeconomic level of society [14]. During the civil wars in Burundi, Rwanda, and Zaire in the 1990s, the prevalence of lice infestations reached 90–100% [15]. The body louse is the species known to be involved in epidemics of louse-borne infectious diseases [16], but all the three cause pediculosis which is highly contagious and easily transmitted by close body-to-body contact or contact with infested linen, brushes, or clothes, according to the species of louse. A louse-infested person can be infested by thousands of lice, each of which bites on average five times per day for body lice [17]. In literature, several methods were used to get rid of lice infestations. Thus, this review summarizes the management methods and various strategies used in treating these hematophagous parasites.

### 1.2. Physiology and Exponential Multiplication of Lice

Humidity is a critical factor for lice; the optimal humidity for survival is in the range of 70–90% [9]; they cannot survive when this value falls below 40%. Temperature is also highly influential on the louse's physiology. According to Maunder, laboratory lice prefer a temperature between 29 and 32°C [9]. At 50°C, body lice die, and this temperature is critical when washing clothes, as water and soap alone will not kill lice [43]. Although eggs can survive at lower temperatures, their viability is limited to 16 days [43].

A louse typically feeds five times a day and each female can have several successive partners. At maturity, lice can mate every day and each female lays 8–10 eggs per day, with a female able to lay up to 300 eggs during her lifetime. During the prolonged mating process, both the male and the female will continue to feed [9]. Eggs are laid in the folds of clothing for body lice and in the hair for head lice; they are held in place by an adhesive produced by the mother's accessory gland [13]. Hatching occurs 7–10 days after laying. There are three nymphal stages (L1, L2, and L3), moulted the third, fifth, and tenth days after hatching and which are differentiated by their size.

### 1.3. Remarks on the Genetics of the Louse (Body Lice versus Head Lice) and Its Symbiont

Genetic tools were used to separate human lice into head lice and body lice. The first study was based on the 18S rRNA gene [44], and subsequent studies focused on mitochondrial genes [45–47] and intergenic spacers [48, 49]. These studies revealed that there are three clades of head lice, one of which may also be body lice (clade A) [46, 47]. Subsequently, a fourth clade including both head and body lice was detected in Congo [50]. A transcriptome study of human head and body lice revealed that there is only one gene present in body lice but not in head lice. Otherwise, the main differences identified between head lice and body lice concern gene expression levels [51]. Indeed, 14 putative differentially expressed genes were identified by comparing head louse and body louse data. However, head lice and body lice have almost the same genomic content but are phenotypically different (different ecotypes) as a result of differential gene expression [52]. Thus, a rapid multiplex real-time PCR assay was established that differentiates between head and body lice with 100% specificity and sensitivity [53]. Based on these studies, we can suggest that the clade A head louse has a deleted genome and originated from the body louse. The opposite hypothesis was considered evident for years [9, 45, 54].

The primary endosymbiont of human body lice is a bacterium belonging to the family Enterobacteriaceae in the*γ-*Proteobacteria class [5]. Organs called mycetome host the primary endosymbiont, except during passage to the ovaries for transovarial transmission [55]. A recent study of the genome of* Candidatus* Riesia pediculicola revealed a small genome, 574 kB, similar to what is found in other insect primary endosymbionts [56]. The reduction in genome size and the high AT-bias suggest an ancient association between the louse and its primary endosymbiont [56]. Thus,* Candidatus* Riesia pediculicola is an insect primary endosymbiont (P-endosymbiont) that has been associated with the louse for 13–25 million years [52].

### 1.4. Louse-Borne Infectious Diseases

Louse-borne diseases are associated with a high prevalence of body louse infestation and have recently reemerged in jails and refugee camps in central and eastern Africa [15], in rural communities in the Peruvian Andes [57], in rural populations in Russia [58], and in homeless populations living in poor hygiene conditions in developed countries [59–63]. Given the phagocytic activity of their immune system, it is more likely that body lice are vectors of pathogens than head lice [64]. The physiological difference between head and body lice is that head lice do not transmit human diseases, whereas body lice are vectors of bacterial diseases transmitted to humans, including trench fever caused by* Bartonella quintana*, relapsing fever caused by* Borrelia recurrentis*, and epidemic typhus caused by* Rickettsia prowazekii* [1, 57, 65].* Acinetobacter baumannii* was found in 21% of the 622 body lice collected worldwide [66], but the transmission of the infection* A*.* baumannii* by body louse has not yet been demonstrated. In Morocco in the 1940s, the causative agent of plague,* Yersinia pestis*, was recovered from a body louse collected from a septicemic patient [67, 68]. In the Democratic Republic of the Congo, DNA from* Yersinia pestis* was observed in one head louse and two body lice [50, 69]. In addition, the experimental model to evaluate the human body louse as a vector of plague has been demonstrated in our laboratory [70].

Head lice can carry pathogens although their role as a vector has not yet been clarified. DNA from* B. quintana* was found in head lice from Nepalese children in 2006 [71], from homeless individuals in the USA in 2009 [65], and from the local population in Congo, Madagascar, and Senegal [72]. It was also found in head louse nits from a homeless person in Marseille, France [73], and in head lice from Ethiopian [74] and Senegalese [75] patients. DNA from* A*.* baumannii* was detected in 33% and 3% of the head lice collected from schoolchildren in Paris, France [76], and Diankabou, Mali, respectively [77].

## 2. Diagnosis of Lice Infestations

**Figure 1:**
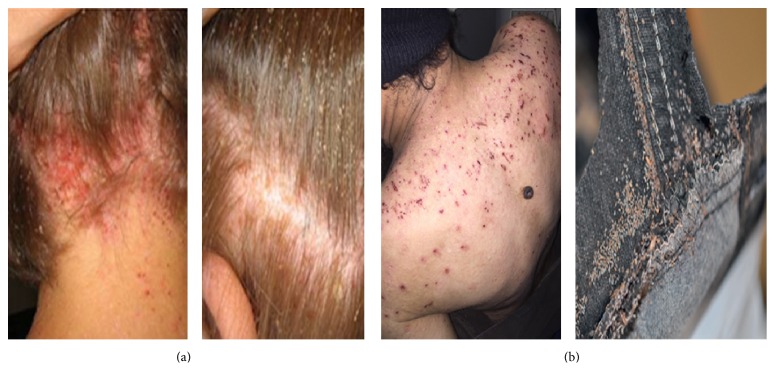
Nuisance related to lice: (a) scalp infection caused by head lice; (b) scraping lesions related to body lice infestation.

**Figure 2:**
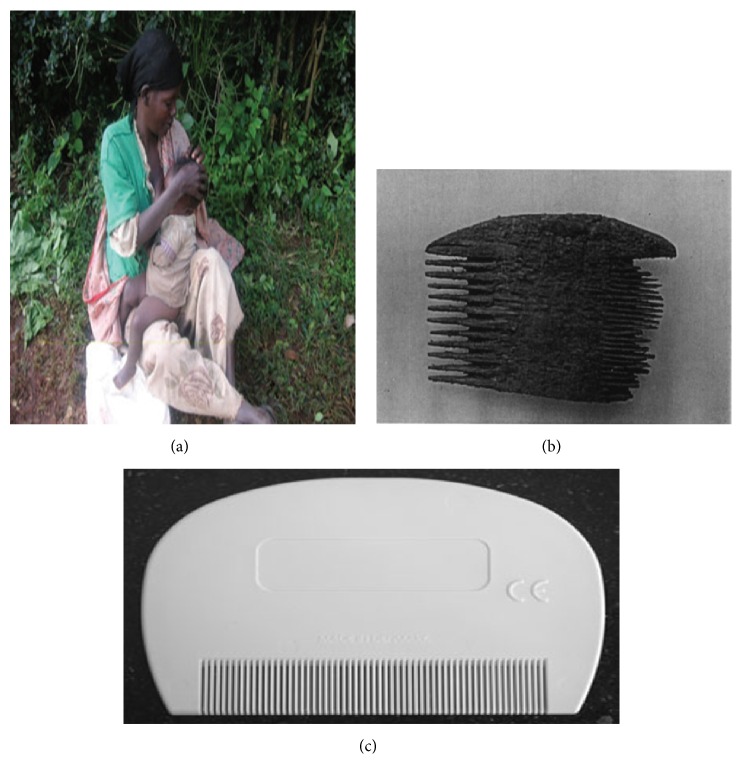
A few historical methods to get rid of lice. (a) Detecting lice or nits by direct visual examination; (b) wooden comb found at Ein Rachel (Negev Desert) (100 BC–200 AD) containing 10 head lice and 5 nits; (c) modern plastic comb.

Lice infestation is a common problem and diagnosis is generally based on the presence of nits or lice. The characteristic itching or pruritus that accompanies infestation may in some cases be complicated by bacterial infections (Figures 1(a) and 1(b)) that occur when the skin becomes excoriated [78, 79]. Lice may be seen on the scalp, in the hair, or on clothing of the infected person. However, the techniques used for diagnosing a louse infestation (*Pediculus humanus*) are a source of controversy. Most epidemiological studies have used direct visual examination (visual inspection). Direct visual examination (Figure 2(a)) with a magnifying glass and combing with louse comb (Figures 2(b) and 2(c)) are two frequently used methods; but the first is not a reliable method for diagnosing living lice on hair [80]. It underestimates active infestation and is only useful with heavily infested patients. Elsewhere, Balcioglu et al. have demonstrated in their study that plastic detection comb is better than visual screening for diagnosis of head louse infestation [81].

## 3. Treatment Strategies

The fight against pediculosis is certainly a very ancient concern and various methods have been used to get rid of it.

### 3.1. Historical Methods

Removing lice by hand (Figure 2(a)) or with a lice comb (Figures 2(b) and 2(c)) and shaving the scalp were some of the oldest methods of controlling human lice [82, 83].

#### 3.1.1. Using Hands

This was the first means used before the comb was invented. Crushing lice with your fingers should be strongly discouraged because it can lead to bacterial penetration through the cutaneous route. Manual removal of nits (especially the ones within 1 cm of the scalp) can only be recommended after treatment with a product [84]. One study showed that manual removal is less effective than pediculicides and does not improve results even when used in addition to a pediculicide treatment [35].

#### 3.1.2. Using Combs

Combs for the removal of adult lice and nits have been used since ancient times [82]. Today, many different types of combs are sold to control lice. Combing can be undertaken every 1–3 days. This method not only is for treatment but can be for prevention, to remove mature lice which might otherwise lay eggs and perpetuate the life cycle. However, it was also demonstrated that the diagnosis of louse infestation using a louse comb is four times more effective (25% versus 6%) than direct visual examination and twice (57 seconds versus 116 seconds) as fast [80]. This study was proven by Balcioglu and colleagues in 2008 [81]. Compared to phenothrin in clinical trials, the bug busting method is effective for managing head lice infestation [41]. Thus, we can conclude that using a louse comb to screen patients for lice infestation and treating with a pediculicide are effective.

#### 3.1.3. Shaving

Head shaving can be a simple method to remove the lice and eggs. It was noted that short hair does not prevent head lice infestations [78]. However, head shaving should be avoided whenever possible because it is humiliating, especially for girls. Complete shaving of the head generally does eliminate lice and prevents reinfestation but is rarely an appropriate measure to take in response to infestation [85].

#### 3.1.4. Using Heat

A further step is heating infested clothing and bedding with hot water to destroy all stages of lice. The heat necessary for the destruction of both lice and nits is 52°C for 30 minutes [86]. This method can be easily applied to infested clothes using hot water but cannot be used on infested hair. Lice may, however, make themselves heat resistant by a hormonal process. Heat resistance is achieved by lice excreting heat resistant, protective secretion through their outer skeleton. This is part of the natural defense mechanism of lice. When the lice have become heat resistant, they can tolerate very high temperatures (above 100°C) [87].

#### 3.1.5. Chemical Products

Cresol, naphthalene, sulfur, mercury, petroleum, naphthalene, and petrolatum were employed alone or, for some of these products, in combination with oil or vinegar [83, 88]. Due to major adverse effects and/or resistance, all of these methods have now been discontinued.

### 3.2. Local Products

#### 3.2.1. Study Methods: Sensitivity and Resistance

Measurement of the susceptibility or resistance per se may be performed only by using doses of insecticide applied via an inert carrier, and this is the basis of the WHO tests and their variants [89–93]. The majority of studies have used WHO-recommended protocols [94] or modified versions of those techniques. Studies have sought to measure the effectiveness of pediculicides in controlled laboratory test systems. Measurement of sensitivity to insecticide in formulations cannot be generic and must be measured either* in vivo* by means of a clinical trial or* in vitro/ex vivo* by using the whole formulation [95]. The methodology of* ex vivo* tests conducted by Meinking and colleagues [96, 97], in which the lice were allowed to continuously bubble on wet sponge with pediculicidal and without subsequent washing, may have given unrepresentative results in some cases. Two studies evaluated resistance to permethrin using techniques on the basis of a WHO method employing single concentration applications of insecticide to filter papers and measuring time to death of the test insects, in order to record LT50 and LT95 [94]. However, in one of these studies the levels of insecticide used to measure the lethal dose were considerably lower than those recommended by WHO [98], so in some cases the measure may have been more of vigor tolerance (reduced sensitivity of an insect population due to elimination of the least robust insects by weak selection) than resistance [99]. Tests using laboratory-reared body lice may be less discriminatory than tests employing wild-caught head lice, and they do not identify variations of effectiveness likely to arise as a result of selection pressure [96]. Observations of lice that survived adequate clinical applications of insecticide products containing more than 13% of monoterpenes suggest they have already become resistant to these chemicals [95].

#### 3.2.2. Pediculicides

Since World War II, many insecticides have been used against lice. Among those for treatment of head and body lice were organochlorines (DDT, lindane), organophosphates (malathion), carbamates (carbaryl), pyrethrins (pyrethrum), and pyrethroids (permethrin, phenothrin, and bioallethrin) [82]. Most of these pediculicides were tested in clinical trials to assess their effectiveness and safety (Table 1). However, their pediculicidal and ovicidal efficacy may vary by product components (Table 2).

*(1) Dichlorodiphenyltrichloroethane (DDT) and Lindane*. Organochlorines (DDT and lindane) were the first of the synthetic organic insecticides used. The development of DDT during the 1940s had enormous impact. It was immediately used to dust prisoners of war to control body lice and won wide acceptance, not only for use on humans but also for animals [100]. Lindane has been available since 1951. However, its effectiveness has been compared to other products [33, 34]. Physiological resistance among both head and body louse populations to lindane is widespread [101]. These two organochlorides are neurotoxic for parasites. Due to developed resistance [91] and safety concerns, the use of these products should be discussed [6].

*(2) Malathion*. An organophosphorous insecticide formulated in concentrations of 1.0% and 0.5%, malathion, has been widely used in the USA and Europe. It worked rapidly against adult lice and was usually effective ovicide [102]. Its effectiveness has been tested in clinical trials [31, 32, 42]. However, resistance of body lice to malathion has been reported in Burundi [103] and Ethiopia [104]. In France, head lice resistance to malathion is reported to be based on clinical failure to control infestations [105]. In addition, a randomized study in 22 volunteers found no evidence that malathion was dangerous in the treatment of head lice when the products were applied in accordance with the instructions for use [106]. However, its use in children under 6 months should be avoided [6].

*(3) Carbaryl*. Used since 1976, more recent reviews have reported carbaryl to be less effective than previously thought [107]. Potentially carcinogenic in rodents, its prescription was restricted in UK [6].

*(4) Natural Pyrethrins or Synthetic Pyrethroids (Phenothrin)*. These pyrethroids are closely related to permethrin and are combined with a synergist (piperonyl butoxide) or nonsynergist insecticide (permethrin) [6]. Like malathion, these products can be a fire danger, and burns have been reported [108]. As with permethrin, resistance to this compound has already appeared in France [109], UK [110], and the Czech Republic [111]. In clinical trials, phenothrin has been demonstrated to also be more effective than wet-combing [21]. It seems likely that resistance to pyrethroids will develop much more rapidly than was the case with older compounds [112]. However, many studies worldwide have already described resistance to pyrethrins and pyrethroids [91, 99, 113].

*(5) Permethrin*. Synthetic pyrethroid, introduced for the first time in the 1986s, 1% permethrin, was approved and was available over the counter for use in 1990 [114]. It is one of the most frequently used treatments against human ectoparasites (head lice and scabies) among lindane, malathion, and carbaryl [34, 115]. However, resistance to permethrin has been reported in many studies throughout the world [116–120]. In clinical trial, permethrin compared to soya oil-based shampoo, coconut, and anise in spray has been less effective [39, 40].

*(6) Dimethicone*. Some studies found dimethicone to be a safe product and more effective than permethrin [121]. In their randomized controlled trial, Burgess et al. confirm efficacy of a single application of 4% dimeticone liquid gel in comparison with two applications of 1% permethrin [23]. Used in many clinical trials, its effectiveness may depend on product [24–27, 122]. However, resistance to dimethicone is unlikely due to its physical mode of action [114].

*(7) Oxyphthirine*®. The Oxyphthirine lotion in a single application in treatment of human lice infestations was revealed to be the safest as it was shown to be nonflammable. The product showed a high efficiency (100%) and certain ability to remove attached nits [123]. The lotion, a patented meta-emulsion suitable for treatment of human head lice (*Pediculus capitis*), has a mechanical action that asphyxiates lice and nits [123].

*(8) Benzyl Benzoate/Benzyl Alcohol*. In concentrations of 10% to 30%, this substance has been widely used for treatment of pediculosis and scabies, although it is not always effective ovicide. This product may cause allergic reactions and skin irritations. It is no longer registered for lice control in the USA, and in Canada it is only available on prescription [112]. Benzyl alcohol 5% is nonneurotoxic and kills head lice by asphyxiation. Its side effects are pruritus, erythema, pyoderma, and ocular irritation [114].

*(9) Spinosad*. Spinosad 0.9% topical suspension is a new ovicidal and pediculicidal treatment against head lice created by fermenting* Saccharopolyspora spinosa*, a bacterium found in the soil [124]. However, the most common adverse reactions observed include erythema, ocular irritation, and irritation at the application site [125].

*(10) Other Products*. 1,2-Octanediol, cocamide diethanolamine lotion (cocamide DEA), and tocopheryl acetate 20% were tested in clinical trials against head lice to assess their efficiency and safety (Table 1) [18–20, 22]. However, the adverse effects were reported with 1,2-octanediol and cocamide diethanolamine except tocopheryl acetate. Thus, continued study is recommended to establish long-term safety of new and alternative agents [126].

### 3.3. Per Os Treatment

 As all other external treatments, per os treatment may have more secondary effects.

#### 3.3.1. Ivermectin

Macrocyclic lactone, ivermectin, is widely used throughout central and western Africa as a microfilaricide to control the transmission of human onchocerciasis and Bancroft filariasis and has been effective against ectoparasites and nematodes in veterinary medicine. It is similar to macrolide antibiotic agents but without antibacterial activity. Clinicians have explored using ivermectin for human ectoparasitics, specifically for head lice (and scabies). In clinical trials, its effectiveness has been compared to other products [29, 30]. Ivermectin appears to provide encouraging results in the treatment of head lice [127] with a need for further doses [128], despite a potential resistance to this molecule being demonstrated in laboratory conditions [129]. However, in a cohort of homeless subjects in Marseilles, France, a prevalence of 85% for body lice was observed [130]. This infestation was reduced temporarily to 19% with three doses of oral ivermectin administered at seven-day intervals [130]. Currently, resistance to ivermectin has been widely demonstrated in many arthropods [131, 132] and is an increasing problem for ectoparasite and nematode control. In clinical trials, oral ivermectin, given twice at a 7-day interval, may be more effective [28] or as efficacious [29] as topical 0.5% malathion lotion.

#### 3.3.2. Cotrimoxazole

Although this product (trimethoprim/sulfamethoxazole, TMP/SMX) has also been reported as a treatment in head lice [133], these compounds are not currently recommended for controlling body lice. Moreover, using this molecule as a pediculicide was stopped due to the multiple adverse effects (nausea, vomiting, rash, transient pruritus, and allergic-type reactions) recorded in participants in clinical trials [37].

## 4. New Approaches

The posttreatment reemergence of lice is common and still remains a real challenge. Treatment success depends on improving our knowledge of the fundamental biology and physiology of the louse.

It is important to notice that per os treatment may have more secondary effects as all other oral medicines than external products.

### 4.1. Symbiotic Treatment

**Figure 3:**
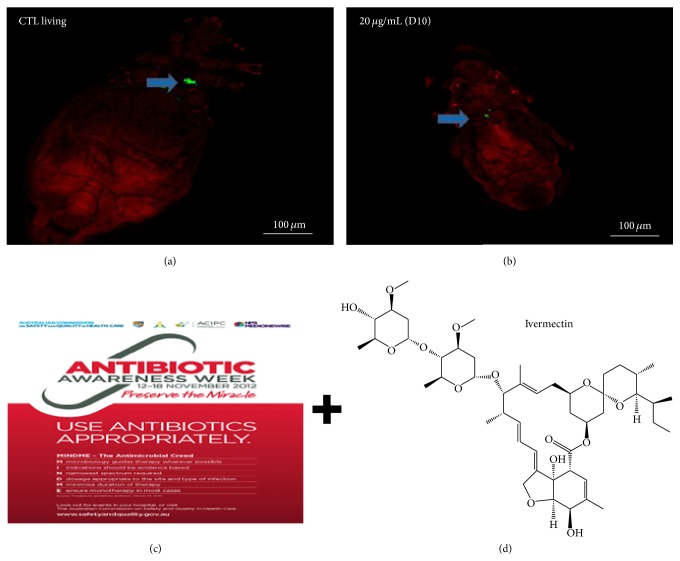
New approaches to get rid of lice:* symbiotic treatment* ((a) living control showing higher bacterial fluorescence; (b) louse treated with doxycycline 20 *μ*g/mL taken at day 10 showing lower bacterial fluorescence) and* synergistic treatment* ((c) antibiotics; (d) ivermectin: chemical structure).

Only a few P-endosymbionts have been described among 14,000 species of hematophagous insects [134, 135]. The endosymbiont* Candidatus* Riesia pediculicola (Figure 3(a)) is a microorganism hosted by body (*Pediculus humanus corporis*) and head lice (*Pediculus humanus capitis*) that appears to be essential for the production of nutritional components such as the B vitamins lacking in host feeding [3, 4]. In our laboratory, we developed an experimental* in vitro* feeding model using an artificial membrane to demonstrate that doxycycline (an antibiotic belonging to the family of tetracyclines) given at different doses (10, 20, and 50 *μ*g/mL) daily for up to 10 days affects the endosymbiont of lice (Figure 3(b)) and also decreases egg production [136]. It was demonstrated that the symbiont* Candidatus* Riesia pediculicola is a possible target for the development of louse-control strategies [56], because loss of these bacteria would mean the death of their hosts. However, symbiotic treatment remains promising and it would be interesting to evaluate the effectiveness of other drugs alone or in combination on lice by targeting their endosymbiont bacterium.

### 4.2. Synergistic Treatment (Antibiotics + Ivermectin): Figures 3(c) and 3(d)

Several compounds, including antibiotics, have been shown to increase intracellular concentrations of macrocyclic lactones [137]. Indeed, antibiotics such as doxycycline, erythromycin, or azithromycin were recommended to treat some infections linked to lice [138]. In addition, it was shown that drug combinations including ivermectin provide antifilarial activity with ancillary benefits on intestinal helminths and ectoparasites, such as chiggers and lice [139]. However, in experiments with adult worms, when doxycycline was combined with macrocyclic lactones in ivermectin the effectiveness was approximately 80% versus 9% for treatment with doxycycline alone [140, 141]. The effectiveness of this combination has also been confirmed in naturally infected dogs with* Dirofilaria immitis* [142]. Recently, in our laboratory we have demonstrated the effectiveness of drug combinations (especially doxycycline + ivermectin, erythromycin + ivermectin, rifampicin + ivermectin, and azithromycin + ivermectin) on body lice reared on rabbits in our laboratory [143]. Thus, we conclude that the synergistic effect is one of the most effective means of lice treatment and also prevents reemergence and resistance.

## 5. Future Efforts

Lice have been intimately associated with humans for centuries. Infestations are increasing worldwide due to insecticide resistance. To date, viable alternative treatments to replace insecticides have been developed experimentally* in vitro*. However, it will be interesting to develop these methods* in vivo* in other studies in order to achieve the complete eradication of lice and avoid the selection of a resistant population of lice. Thus, future efforts should be directed toward the development of pediculicides based on new chemicals such as avermectins and antibiotics.

## Figures and Tables

**Table 1:** Main products used in clinical trials in humans: efficacy and safety.

Comparison of treatments	Efficacy	Safety	References
1,2-Octanediol versus *malathion*	More effective	Adverse effects reported	[18]
1,2-Octanediol versus *placebo*	Effective	No serious adverse events	[19]
Cocamide DEA versus *permethrin*	May be as effective	Adverse effects reported	[20]
Phenothrin versus* wet-combing*	May be as effective	No evidence of harms from combing	[21]
Tocopheryl acetate versus *permethrin*	More effective	No adverse effects reported	[22]
Dimeticone versus *permethrin*	More effective	No serious adverse events	[23, 24]
Dimeticone versus *malathion*	More effective	No adverse effects reported	[25]
Dimeticone versus *dimeticone plus nerolidol*	As effective	No adverse effects reported	[26]
Dimeticone 4% lotion versus *phenothrin*	Equally effective	Few adverse effects reported	[27]
Ivermectin versus *malathion*	As effective	No major adverse effects observed	[28, 29]
Ivermectin versus *placebo (vehicle)*	More effective	No adverse effects reported	[30]
Malathion lotion versus *phenothrin*	More effective	No adverse effects reported	[31]
Malathion versus *permethrin*	More effective	No adverse effects reported	[32]
Lindane versus *permethrin*	As effective	Adverse effects reported	[33]
Permethrin versus *lindane*	More effective	No adverse effects reported	[34]
Permethrin versus *combing*	More effective	No adverse effects reported	[35]
Permethrin versus *placebo*	More effective	No adverse effects reported	[36]
TMP-SMX plus permethrin versus *permethrin alone*	More effective	No major adverse effects reported	[37]
Combined insecticides versus *herbal oils*	As effective	No clinically detectable adverse effects	[38]
Soya oil-based shampoo versus *permethrin*	More effective	No serious adverse events	[39]
Coconut and anise in spray versus *permethrin*	More effective	Adverse effects reported	[40]
Combing versus* phenothrin*	More effective	No evidence of harms from combing	[41]
Bug Buster kit versus *malathion or permethrin*	More effective	No information on adverse effects	[42]

**Table 2:** Main formulations of physical acting and insecticide-based products available in France.

Physical acting products	Principal component(s)	Insecticide-based products	Principal component(s)	Activity
Pouxit® XF Extra Fort	Dimeticone-1.6, dodecatrien-3-ol 3,7,11-trimetyl PEG/PPG dimeticone co-polymersilica silylate	Prioderm®	Malathion	100% pediculicidal and ovicidal activity
Duo LP Pro®	Triglycerides, lipid esters (Oxyphthirine)			

Itax®	Oily silicone based complex			100% pediculicidal activity and insufficient ovicidal activity
Altopou®	Cyclomethicone 5, dimeticone	Marie-Rose	Pyrethrin	
Pouxit	Cyclomethicone 5, dimeticone	Para® Special Poux	Alethrin (prallethrin)	
Paranix® mousse	Dimeticone, paraffin oil	Para Plus	Malathion, permethrin	
Paranix new formule action double	Dimeticone, mineral oil			
Marie-Rose® une seule application	Cocamidopropyl betaine cocamide DEA			

Parasidose® lotion traitante	Ricinus, paraffin, cocamide DEA, cocos	Pyreflor® lotion	Permethrin 25/75	Insufficient pediculicidal activity and insufficient ovicidal activity
Parasidose nouvelle formule Biococidine®	Biococidine	Sklice® lotion	Ivermectin	
Yapapou®	*Cocos nucifera*, cocamide DEA, citric acid, cocamidopropyl			
Poux Apaisyl®	Coconut oil derivatives			
