# Expression of Concern on “Management and Treatment of Human Lice”

**DOI:** 10.1155/bmri/9870486

**Published:** 2026-06-02

**Authors:** BioMed Research International

EXPRESSION OF CONCERN: A. K. Sangaré, O. K. Doumbo, and D. Raoult, “Management and Treatment of Human Lice,” BioMed Research International 2016 (2016): 8962685, 10.1155/2016/8962685.

This Expression of Concern is for the above article, published online on 27 July 2016 in Wiley Online Library (wileyonlinelibrary.com), and has been issued by agreement between the journal Editor‐in‐Chief, Yoel Klug; and John Wiley & Sons Ltd. The Expression of Concern has been agreed due to questions about the research upon which this literature review is based. Complaints have been raised publicly on https://pubpeer.org/ for nine of the referenced articles, most of which involve vulnerable populations:

1. Benkouiten et al., 2014: 10.1001/jamadermatol.2013.6398
2. Sangaré et al., 2016: 10.1016/j.ijantimicag.2016.01.001
3. Piarroux et al., 2013: 10.3201/eid1903.121542
4. Foucault et al., 2006: 2006:10.1086/499279
5. Drali et al., 2015: 10.4269/ajtmh.14-0686
6. Drali et al., 2013: 10.1371/journal.pone.0058088
7. Drali et al., 2012: 10.1128/jcm.00808-12
8. Angelakis et al., 2011: 10.1111/j.1574-695X.2011.00804.x
9. Veracx et al., 2012: 10.1371/journal.pone.0037804

Additionally, concerns of a potential conflict of interest have been raised on PubPeer regarding author Didier Raoult. The investigation into these concerns is ongoing. Therefore, the journal has decided to issue an Expression of Concern to inform and alert readers.

